# Gestational Diabetes Insipidus Associated with HELLP Syndrome: A Case Report

**DOI:** 10.1155/2012/640365

**Published:** 2012-07-09

**Authors:** Renela Gambito, Michael Chan, Mohamed Sheta, Precious Ramirez-Arao, Harmeet Gurm, Allan Tunkel, Noel Nivera

**Affiliations:** ^1^Department of Internal Medicine, Monmouth Medical Center, Long Branch, NJ 07740, USA; ^2^Section of Nephrology, Monmouth Medical Center, Long Branch, NJ 07740, USA

## Abstract

Gestational diabetes insipidus is a rare, but well recognized, complication of pregnancy. It is related to excess vasopressinase enzyme activity which is metabolized in the liver. A high index of suspicion of gestational diabetes insipidus is required in a correct clinical setting especially in the presence of other risk factors such as preeclampsia, HELLP syndrome, and twin pregnancies. We are presenting a case of gestational diabetes insipidus in a patient with HELLP syndrome. The newborn in this case also had hypernatremia thereby raising possibilities of vasopressinase crossing the placenta.

## 1. Introduction

Gestational diabetes insipidus is a rare complication of pregnancy, and it is related to excess vasopressinase activity. Measurement of the vasopressinase serum concentrations is not available commercially, and the diagnosis is usually established based on the clinical presentation and the appropriate laboratory studies. We are describing a unique case of gestational diabetes insipidus in a patient with HELLP syndrome. Both mother and infant presented with hypernatremia and dehydration.

## 2. Case Report

A previously healthy 47-year-old woman in her 33rd week of twin gestation presented with progressive lethargy, confusion, polydipsia, and polyuria for approximately one month. She was only on prenatal vitamins, and her prenatal follow-up was unremarkable. On admission, her blood pressure was 108/60 mmHg, pulse 95 beats/minute, temperature 97°F, respiratory rate of 17 breaths/min, and saturating 98% on ambient air. Her physical examination was significant for confusion, dry oral mucosa, and gravid abdomen. However, there were no petechial lesions, lower limb edema, or focal neurological deficit. Her cardiac and pulmonary examination was also normal.

Laboratory studies revealed a serum sodium of 171 mEq/L, potassium 4.1 mEq/L, chloride 138 mEq/L, bicarbonate 21 mEq/L, glucose 197 mg/dL, BUN 49 mg/dL, creatinine 3.2 mg/dL, calcium 7.8 mEq/L, total protein 5.4 gm/dL, albumin 1.6 gm/dL, alkaline phosphatase 576 units/L, AST 391 units/L, and ALT 443 units/L. The serum osmolality was 356 mOsm/kg; urine sodium 21 mEq/L and urine osmolality was inappropriately low (225 mOsm/kg). Urinalysis was significant for a specific gravity of 1.008, red blood cells 8–10/high-power field, and 30 mg/dL of protein. Complete blood count showed hemoglobin of 13.7 g/dL, hematocrit 42.6%, white blood cell count 15,200/mm^3^, and platelet count of 76,000/mm^3^.

The patient underwent an emergent cesarean section, which was complicated by the demise of one of the twin babies. The other newborn was a female with a birthweight of 2180 grams. APGAR scores were 0, 4, and 7 at one, five, and ten minutes, respectively. Laboratory evaluation of the infant showed serum sodium of 177 mEq/L, potassium 4.4 mEq/L, chloride 140 mEq/L, bicarbonate 14 mEq/L, BUN 49 mEq/L, creatinine 3.2 mg/dL, glucose 30 mg/dL, and calcium 8.3 mEq/L.

The infant received 2 boluses of 10% Dextrose for hypoglycemia and was then placed on Dextrose 10% in water at 6 mL/hour with normal saline at 1 mL/hour via umbilical vein. The hypernatremia and the acute kidney injury corrected over a course of several days with continued fluid administration. Her serum antidiuretic hormone level, at the time of birth, was <0.5 pg/mL (Normal 0.0–4.7 pg/mL).

**Figure 1:**
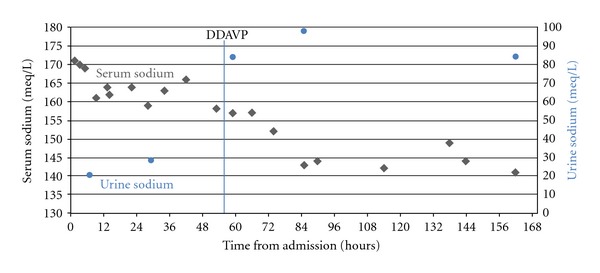
Serum and urine sodium before and after DDAVP administration.

**Figure 2:**
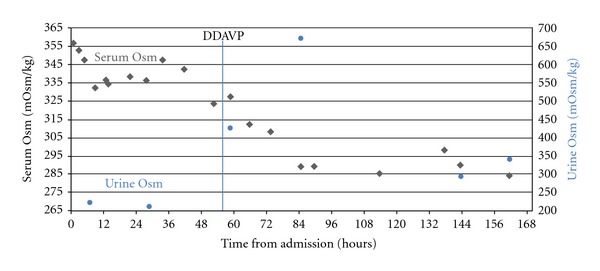
Serum and urine osmolality before and after DDAVP administration.

Postoperatively, the mother continued to be hypernatremic despite IV infusion of hypotonic fluids. On the third day postpartum, the diagnosis of gestational diabetes insipidus was suspected and the patient was started on intranasal DDAVP spray 5 mcg twice a day, after an initial dose of 10 mcg. Marked improvement was seen in the patient's hypernatremia, in which her serum sodium concentrations decreased from 158 mEq/L to143 mEq/L within 30 hours from the initial DDAVP dose (Figure 1), and normalization of her serum osmolality (Figure 2). In addition, clinically she became more alert.

## 3. Discussion

Gestational diabetes insipidus is a rare complication of pregnancy that occurs in 4 out of 100,000 pregnancies as discussed by Durr [1]. Symptoms usually develop over a few days and include polyuria, polydipsia, fatigue, nausea, weight loss, and decreased skin turgor. Its pathophysiology is thought to result from increased degradation of arginine vasopressin (AVP) by a placental enzyme called vasopressinase.

Vasopressinase is a cysteine aminopeptidase of molecular weight 330 kDa, and is produced by the placental trophoblasts during pregnancy as discussed by Gordge et al. [2]. Serum concentrations can be detected as early as the 7th week of gestation, reaching the maximum around the 40th week, and are usually undetectable around the 6th week postpartum as described by Davison et al. [3]. Being synthesized from the placenta, higher enzyme concentrations were observed in twin and triple pregnancies.

The primary known action of vasopressinase is *in vivo *as well as *in vitro *degradation of vasopressin by removing amino acids from the N-terminus. Since the exogenous desmopressin is deaminated at the N-terminus, it can escape the degrading effect of the vasopressinase and is considered the treatment of choice in patients with gestational diabetes insipidus as demonstrated by Schrier [4]. Vasopressinase is metabolized in the liver, which may explain higher concentrations of the enzyme in patients with fatty liver, hepatitis and preeclampsia as discussed by Barbey et al. [5].

HELLP syndrome is considered a spectrum of preeclampsia. The pathophysiology is not well understood but has been hypothesized to result from excessive stimulation of V1 receptors by vasopressinase-altered AVP leading to platelet aggregation, vasospasm, endothelial constriction, and brain stimulation as discussed by Krege and Katz [6]. On the other hand, liver dysfunction in patients with HELLP syndrome decreases the degradation of the vasopressinase as described by Ellidokuz et al. [7].

In our case, the infant was hypernatremic with high serum osmolality and a relatively low ADH level (<0.5 pg/mL). Therefore, it is possible that vasopressinase could have crossed the placenta thereby causing infant's hypernatremia and subsequently could have benefited from dDAVP.

In light of the above case, we recommend maintaining a high index of suspicion of gestational diabetes insipidus in patients who present with typical symptoms and signs especially in the presence of other risk factors such as preeclampsia, HELLP syndrome, and twin pregnancies. In addition, treating the infant with DDAVP besides intravenous fluids may also be beneficial.
